# Mass AITC Supports Technologies Incorporating Machine Learning and AI to Support Successful Aging and Care of PLwD

**DOI:** 10.1093/geroni/igaf122.1524

**Published:** 2025-12-31

**Authors:** Niteesh Choudhry

**Affiliations:** Harvard Medical School, Boston, Massachusetts, United States

## Abstract

MassAITC supports pilot studies on using mobile technologies, wearables, and contactless sensors that incorporate emerging machine learning and AI approaches to support successful aging and the care of individuals with ADRD in their home environments. We will provide an overview of studies supported by our Center, which have addressed a broad range of topics including the early diagnosis of cognitive impairment, cognitive rehabilitation, longitudinal monitoring of chronic conditions of aging, falls and safety detection, balance and mobility training, cardiovascular risk assessment and management, and caregiver support. We will describe some of the early successes resulting from these awards including the formation of new start-up companies (e.g. Billion Labs Inc.), company acquisitions (e.g. Kinto by Rippl and TellUs You Care by AIP Healthcare), venture capital funding (e.g. Butlr and Moneta Health), capital and business alliances (e.g. Butlr and Ricoh), and design innovation awards (e.g. MindMics). We will also discuss some of the unique challenges arising from the funding of predominantly industry investigators within the context of a NIH granting mechanism. These have included the timelines and requirements for regulatory oversight, including IRB approvals, the management of conflicts of interest and intellectual property interests, the need to provide mentorship about study design and the conduct of human subjects research, difficulties recruiting individuals with ADRD for investigators not working in environments without an established pool of potential participants, and limitations of the amount of data available to build ML/AI models within the constraints of the size of the individual awards.

